# Evaluating the role of graft angle on cerebral hemodynamics following direct cerebral bypass for moyamoya disease

**DOI:** 10.1371/journal.pone.0330362

**Published:** 2026-01-05

**Authors:** Cheng Peng, Ephraim W. Church, Melissa C. Brindise

**Affiliations:** 1 Department of Mechanical Engineering, Pennsylvania State University, University Park, Pennsylvania, United States of America; 2 Departments of Neurosurgery, Neurology, and Radiology, Penn State Health, Hershey, Pennsylvania, United States of America; Coventry University, UNITED KINGDOM OF GREAT BRITAIN AND NORTHERN IRELAND

## Abstract

Direct cerebral bypass is a key treatment for moyamoya disease (MMD). This surgery grafts a donor vessel onto a recipient cerebral artery to boost blood flow to hypoperfused brain regions. Unlike coronary bypass, which restores downstream flow around a blockage, cerebral bypass for MMD reverses flow in the recipient vessel to perfuse the upstream network. Surgical decisions—such as donor vessel choice and anastomosis angle—significantly affect graft hemodynamics and outcomes. Yet these choices still rely on neurosurgeons’ experience, lacking quantitative guidance. This study addresses that gap by examining how anastomosis angle shapes post-surgical perfusion and wall shear stress. We created idealized cerebral bypass models with graft angles of 30°, 60°, and 90°. Each model’s flow field was assessed under varying inflow and graft-flow combinations using computational fluid dynamics. The 30° angle produced the strongest reverse flow but also the largest WSS imbalance, potentially driving long-term complications. The 60° angle achieved adequate reverse flow with a more uniform WSS profile, making it the most favorable. Overall, our results show graft angle must be carefully considered in cerebral bypass planning.

## Introduction

Moyamoya disease (MMD) is characterized by reduced blood supply to the brain due to the narrowing of cerebral arteries [1]. This narrowing prompts the proliferation and enlargement of small vessels, increasing the risk of ischemic events and strokes [2]. While the incidence of MMD was 0.086 per million in the millennium, it has significantly increased in recent decades [3,4]. MMD is associated with a significant annual stroke risk and is the leading cause of stroke in children in the United States [5,6].

**Fig 1:**
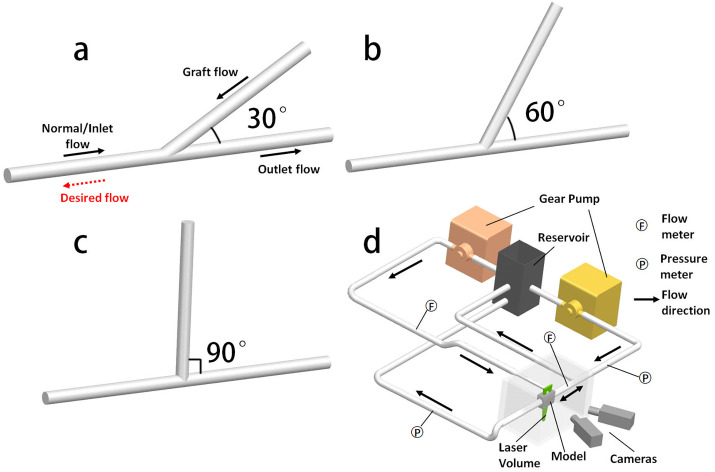
The schematic of the bypass models with 30°(a), 60°(b) and 90°(c) graft angles. Ideally, the normal inlet flow should be reversed by the higher graft flow to increase overall perfusion. (d) The schematic of the flow loop. Notice the normal inlet was designed to be freely reversed.

Direct cerebral bypass surgery is widely acknowledged as the most effective method for treating MMD and related conditions that cause cerebral hypoperfusion [7–12]. Traditional bypass surgeries, such as coronary artery bypass grafting (CABG), aims to restore unidirectional blood flow by creating alternative pathways around blocked vessels. In contrast, direct cerebral bypass surgery for MMD involves grafting from extracranial to intracranial arteries—typically, using the superficial temporal artery (STA) to the middle cerebral artery (MCA)—in order to reverse the flow in the intracranial (i.e., MCA) artery (see Fig 1a) and enhance perfusion throughout the entire vascular tree. Hence, the graft vessel must deliver a higher flow rate than that which exists in the recipient vessel.

However, it is important to note that while the goal is to reverse perfusion in the recipient vessel, hyperperfusion in the recipient vessel can pose significant risks, including stroke or cognitive decline [13,14]. Thus, these risks necessitate meticulous surgical planning. Factors such as the choice of donor and recipient vessels, the anastomosis technique, and the geometry of the anastomosis are expected to critically affect the degree of retrograde perfusion the surgery will induce [1]. However, few fundamental studies have been reported which investigated the specific quantitative impact of these surgical decisions [15,16]. Prior studies evaluating this flow problem have been limited to assessing patient-specific postoperative hemodynamic metrics, [17,18] examining changes in hemodynamic variables across postoperative remodeling states, or predicting long-term complications [19,20]. While such studies provide valuable clinical insight, they are limited in two ways: first, they examine outcomes only after graft implantation and remodeling, making it difficult to isolate the mechanistic role of specific surgical variables; second, patient-specific vascular variability (diameter, tortuosity, collateral status) complicates the interpretation of how one parameter affects hemodynamics. Hence, studies have not systematically explored how preoperative decisions impact the hemodynamics of reverse flow, specifically, the anastomosis angle. As a result, cerebral bypass surgical decisions currently rely solely on the experience of neurosurgeons, rather than objective, physics-based evaluations.

In this paper, we address this critical gap by systematically evaluating how the anastomosis angle affects graft hemodynamics and reversed flow rates across various donor and recipient vessel flow rate combinations. The use of idealized model allows us to directly investigate the mechanistic influence of graft angle under controlled conditions. In this study, we utilized both stereo-particle image velocimetry (PIV) experiments and computational fluid dynamics (CFD) simulations of the bypass models. This dual-modality strategy enabled a more robust assessment of the hemodynamic impact of graft angle than either technique alone: the PTV was used to rigorously validate our CFD model, while the use of CFD enabled us to evaluate more test cases than feasible using PTV alone. Using the flow fields for each model, we evaluated the reversed perfusion percentage induced by the graft vessel as well as the resultant WSS distribution. Generally, this study provides a fundamental reference for direct bypass surgery that could be used to inform surgical decisions and potentially improve patient outcomes in MMD treatment.

## Materials and methods

This study did not involve human participants, human data, or animal subjects. Therefore, approval by an institutional review board (IRB) or ethics committee, and informed consent, were not required.

### Bypass models and flow loop

Three idealized bypass morphologies with graft angles of 30°, 60°, and 90° were fabricated using polydimethylsiloxane (PDMS-Sylgard 184). To manufacture the models (Fig 1a–c), the bypass geometries were first constructed as computer aided design (CAD) models using Siemens NX (Unigraphics). The models had a uniform inner diameter of 2.6 mm and no curvature. The CAD models were 3D printed (LulzBot TAZ 5) in polyvinyl alcohol (PVA) and meticulously polished. The 3D printed models were placed in custom-designed acrylic boxes, and the liquid PDMS, mixed according to the standard 1:10 ratio, was poured into the boxes. The PDMS was cured at room temperature for at least 36 hours. The inner PVA structure was subsequently dissolved using water, resulting in a rigid, hollow, and optically transparent PDMS model.

An *in vitro* flow loop was assembled to simulate *in vivo* conditions (Fig 1d). Because the loop needed to allow for reversed flow of the inlet, a T-fitting outlet was added upstream of the model, so a portion of the inlet flow returned directly back to the reservoir, and part moved through the model. The second pump provided flow to the graft vessel. Steady flow was used. The test matrix included all combinations of inlet flow rates of 0, 3, and 5 ml/min and graft flow rates of 5, 10, 15, 20, and 25 ml/min. These flow rates were selected as the typical ranges for each vessel measured by a neurosurgeon during direct bypass surgeries using a flow probe. Flow rates were measured using a Transonic (TS 410) flowmeter at both the inlet and graft vessels. Pressure transducers were placed at the inlet and outlet of the main vessel. The working fluid was a blood-analog water-glycerol-urea mixture with a viscosity and density of 4.67 cP and 1143 kg/m3. This working fluid ensures the best optical visibility with the closest fluid property compared to blood [21]. Although this introduces modest differences in viscosity and density compared to the CFD model, the Reynolds number in all tested conditions remained well within the laminar regime (Re = 6–47). Hence, the primary flow patterns, velocity fields, and relative wall shear stress distributions are preserved.

### PIV experimental technique

Stereo-PIV images were captured using an Nd-YAG laser (EverGreen, λ = 532 nm) and two high-speed cameras (ImagePro X, LaVision Inc.). The laser thickness was approximately 1 mm and aligned with the center plane of the model [22]. The cameras were positioned with a ± 20° angle off the central line on the same side of the models and calibrated using a dual-plane calibration target with RMS < 0.6. The working fluid was seeded with 10.5 μm fluorescent particles. Images of size 2560 × 2160 pixels were captured using a double pulsed timing sequence with a pair capture frequency of 15 Hz and a time interval (Δt) between the two frames in a pair of 1000 μs. This Δt corresponded to a roughly 10 pixels particle displacement between image pair frames. A camera magnification of approximately 8 μm/pixel was used. The refractive index of the working fluid was matched to the PDMS model (n = 1.4118), and the model was submerged in the same fluid to reduce the optical distortion. The PIV images were processed using Davis 10.0 (LaVision Inc.). Stereo sum of correlation with 150 image pairs were used to calculate the average vector field. Two PIV passes were used, with the first pass using a 48 × 48 pixel, 50% overlap window and the second pass using a 24 × 24 pixel, 75% overlap window as well as 6-pixel grid. This resulted in time-averaged 2D velocity fields of size 427 × 360 vectors. The use of stereo PIV as opposed to planar PIV for 2D data capture provides more accurate velocity fields by better accounting for out of plane velocities.

### CFD simulation technique

CFD simulations were conducted using ANSYS Fluent 2023 R2 to solve the governing Navier-Stokes equations. The fluid was assumed to be incompressible and Newtonian, with a density of 1060 kg/m^3^ and viscosity of 0.0035 kg/(m ∙ s). Due to the low Reynolds number, laminar flow conditions were applied. A rigid wall boundary condition was imposed, and a steady, pressure-based solver was employed. Boundary conditions were set as follows: the inlet was specified as a pressure-inlet, the graft as a velocity-inlet, and the outlet as a pressure-outlet. This configuration allowed for a free reversed inlet simulation.

### Post-processing

Geometry masks were used in Davis and the masked data were exported as the raw velocity field. The time-averaged velocity field was used for all post-processing calculations which induces inherent smoothing; thus, no additional smoothing was done.

The WSS of the top and bottom walls of the inlet vessel (main vessel (MV)) were computed. For this calculation, velocity gradients were calculated as:


$$\begin{array}{c} \frac{du}{dx_{i}}=\;\frac{u_{i+\text{1}}-u_{i}}{x_{i+\text{1}}-x_{i}} \end{array}$$ (1)

$$\begin{array}{c} \frac{dv}{dx_{i}}=\;\frac{v_{i+\text{1}}-v_{i}}{x_{i+\text{1}}-x_{i}} \end{array}$$ (2)

$$\begin{array}{c} \frac{du}{dy_{i}}=\;\frac{u_{i+\text{1}}-u_{i}}{y_{i+\text{1}}-y_{i}} \end{array}$$ (3)

$$\begin{array}{c} \frac{dv}{dy_{i}}=\;\frac{v_{i+\text{1}}-v_{i}}{y_{i+\text{1}}-y_{i}} \end{array}$$ (4)

where u and v are the x- and y-direction velocities. WSS was then calculated as:


$$\begin{array}{c} \tau_{x}=\;\mu \;\left({\text{2}n}_{x}\left(\frac{du}{dx}\right)+n_{y}\left(\frac{du}{dy}+\frac{dv}{dx}\right)\right) \end{array}$$ (5)

$$\begin{array}{c} \tau_{y}=\;\mu \;\left(n_{x}\left(\frac{du}{dy}+\frac{dv}{dx}\right)+\text{2}n_{y}\left(\frac{dv}{dy}\right)\right) \end{array}$$ (6)

$$\begin{array}{c} \tau_{mag}=\;\sqrt{{\tau_{x}}^{\text{2}}+{\tau_{y}}^{\text{2}}} \end{array}$$ (7)

where τ_x_ and τ_y_ are the WSS components in the x- and y-direction, τ_mag_ is the WSS magnitude, μ is the dynamic viscosity, and (*n*_*x*_, *n*_*y*_) is the unit normal vector. The unit normal vectors were defined as the vector perpendicular to the user-specified wall locations. Further data analysis used an open source Violin Plot code based on kernel density estimation [23]. All post-processing was done in MATLAB.

## Results

We conducted a quantitative comparison between the CFD and PIV velocity fields to validate the CFD model against the experimental results. We found our CFD and PIV velocity fields matched to within ~7%, highlighting strong agreement between the two modalities and adequate validation of the CFD. The complete validation analysis is available in the supplementary material S1 File.

### Velocity field distribution analysis

**Fig 2:**
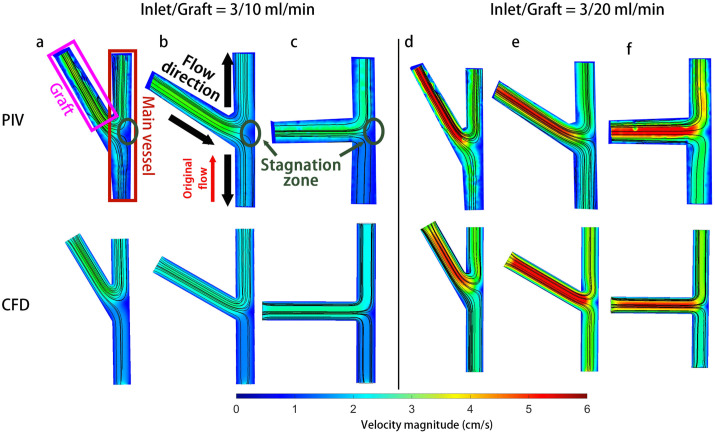
The streamline and velocity contour plot for the PIV and CFD at inlet/graft = 3/10 & 3/20 ml/min for 30° (a & d), 60° (b & e) and 90° (c & f). Note: These are vector fields for two typical cases. The rest of the cases are not shown.

Fig 2 illustrates the velocity magnitude distribution for the cases with inlet/graft flow rates of 3/10 and 3/20 ml/min for both PIV and CFD. As expected, the higher graft flow rate visually produced more reversed flow through the inlet vessel. The most striking effect of the varied graft angle was the size of a stagnant flow region induced at the top MV wall near the anastomosis. This stagnant flow region was observed to increase in size with decreasing graft angle, such that the 30º graft angle produced the largest stagnation zone, while the 90º region produced the smallest one. Across all cases, a consistent flow trend with no vorticity or swirling flow motion was observed, likely owing to the very low flow rate and Reynolds number.

### Backflow perfusion analysis

**Fig 3:**
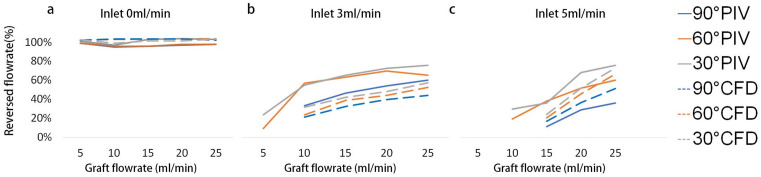
The reversed flowrate portion for all the cases for inlet from 0 (a), 3 (b) and 5 (c) ml/min. The reverse percentage was calculated as the negative (reversed) inlet flow divided by the outlet flow.

Fig 3 evaluates the reversed flowrate percentage in the MV inlet across all tested cases, which was defined as the volumetric flow entering the MV inlet in the reverse direction divided by the volumetric flow exiting through the MV outlet. Thus, 100% indicates equal flow through both the inlet and outlet branches, while 0% indicates no reversal and all flow exited distally through the MV outlet.

Retrograde perfusion remained nearly 99% for all angles when the inlet flow rate is 0 ml/min (Fig 3a). When inflow reached 3 ml/min, only the grafts with angle (30**°** and 60**°**) preserved appreciable back perfusion, reported around 22%, while 90**°** anastomosis failed to produce any backflow. Further increase of the inlet flow steadily restored reversal in the 30**°** and 60**°** grafts, increased from ~60% to ~70%, while the 90**°** angle lagged around 25%. Computational results followed the same angle-ranked pattern, but with uniformly lower magnitudes (15%−20% below experimental values).

At an inlet flow of 5 ml/min (Fig 3c) no configuration produced retrograde perfusion with a 5 ml/min graft flow. When graft inflow reached 10 ml/min, 30**°** and 60**°** grafts began to recover modest backflow (<25%), whereas the 90**°** graft remained ineffective. However, once the graft flow increased from 15 to 25 ml/min, the backflow climbed steadily, from 30% to 74% in PIV, and 21% to 62% in CFD, for 30**°** and 60**°** angles. In contrast, 90**°** angle lagged throughout, never exceeding ~55% of the reversal.

### Wall shear stress

**Fig 4:**
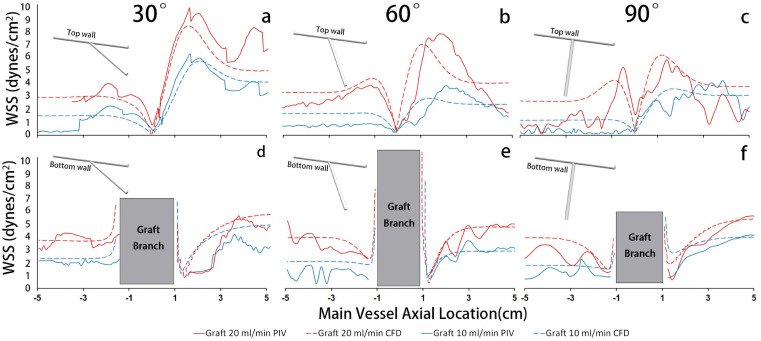
The WSS distribution in the MV for top (a-c) and bottom (d-f) walls, with the same 5 ml/min inlet. + MV represents outlet while -MV represents inlet.

Fig 4a–c shows the WSS distribution at the top wall of the MV for the highest inlet flowrate case (5 mL/min). The outlet (+MV location) WSS was higher than the inlet (-MV location) for all cases. This trend aligns with the observation from Fig 2, where all outlets (-MV location) exhibited higher flow rates than the reversed inlets (+MV locations). In general, the 20 ml/min graft flowrate case yielded higher WSS than the 10 ml/min graft flowrate case, as expected. In the 30° case, the average WSS of the outlet MV was about 7.5 dynes/cm^2^. For the inlet MV, the average WSS was about 3.1 dynes/cm^2^, nearly 110% lower than the outlet MV. A 60**°** graft elevated outlet WSS to ~6 dynes/cm^2^, creating a 70% inlet-outlet mismatch. Increasing the angle to 90**°** reduced such gap slightly outlet WSS stayed around 6 dynes/cm^2^ but inlet WSS climbed to 3.8 dynes/cm, reducing the mismatch to 60%. Notably, a significant WSS drop, registering below 1 dynes/cm^2^, was observed opposite the anastomosis for all cases; this correlates with the low velocity stagnation area depicted in Fig 2.

Fig 4d–f illustrates the WSS distribution at the bottom wall of the MV. Notably, two WSS drops near the junction of the graft branch aligned with the peaks observed in the top walls. This consistency suggests a correlation between top and bottom wall WSS distribution, further supported by Fig 2’s depiction of the bending streamlines. Apart from the drops near the junction, no significant differences in WSS were observed between the inlet and outlet. Specifically, in the 30° configuration, the average WSS ranged from 4 to 4.7 dynes/cm^2^ at the inlet and outlet. In the 60° configuration, the average WSS was 3.9 dynes/cm^2^ at the inlet and 4.5 dynes/cm^2^ at the outlet. Similarly, the 90° configuration exhibited a close average WSS, measuring 4 dynes/cm^2^ at the inlet and 4.9 dynes/cm^2^ at the outlet. Although the CFD produced smoother WSS distributions as expected, both PIV and CFD data demonstrated similar results, with a difference of less than 1 dynes/cm^2^.The CFD simulation highlighted a dramatic increase in WSS adjacent to the graft branch, with WSS intensifying closer to the graft branch. The PIV did not show this same trend. However, computing accurate velocity gradients from the PIV at this point is challenging due to the sharp transition at the intersection points between the graft and the MV. Additionally, the model manufacturing process likely decreased the sharpness of this bend for the PIV data.

## Discussion

Conditions associated with cerebral hypoperfusion such as Moyamoya disease are associated with a significant risk of stroke [2]. Despite extensive evaluation of its genetic and pathophysiological underpinnings, the efficacy of drug treatments remains contentious, particularly concerning the selection of specific antiplatelet agents and their therapeutic outcomes [2,15,24,25]. Direct cerebral bypass remains the principal treatment for MMD. The primary aim of surgical revascularization is to ensure adequate cerebral blood flow and prevent strokes. Thus, the ability to provide sufficient backflow perfusion (stronger backflow) to the entire target vascular tree is considered a crucial aim of the direct bypass operation. In our study, we investigated three different graft angles using both *in vitro* experimental PIV and *in silico* CFD simulation. To the best of our knowledge, this is the first research study to parametrically evaluate the role of graft angle on hemodynamics following direct cerebral bypass surgery.

Our analysis underscores the effectiveness of optimizing a graft angle for enhancing backflow rates. Clear improvements in reversed flow were observed in both the 30° and 60° cases. In particular, our results indicate that the 30° angle provides 10% more backflow than the 60° angle and 30% more backflow than the 90° angle. Given that complications of direct bypass have been associated with high graft flow rates [1,2,11,13,14], our study suggests a novel approach to increasing backflow perfusion without necessitating an increase in graft flow.

The WSS analysis revealed that the MV wall maintained disparate shear stress values outlet and inlet to the graft vessel anastomosis. This finding agrees with fundamental flow expectations given that the outlet and inlet portions also maintained significantly different velocity magnitudes. Moreover, the stagnation region demonstrated in Fig 2 at the anastomosis would be expected to result in high relative residence time (RRT) of blood flow at that point. Because time-averaged velocity fields were used here, RRT could not be explicitly computed to confirm this notion, and this should be explored in future work. Nonetheless, both the non-uniform WSS distribution and stagnation region would be expected to promote post-operative vessel remodeling and potential complications. Altered and non-uniform WSS induces endothelial dysfunction that leads to vascular remodeling aimed at regularizing the WSS distribution. This type of vascular remodeling can lead to aneurysm formation or atherosclerosis [26–29]. Meanwhile, the low velocity and low WSS region stagnation region can facilitate thrombus formation since the slow flow provides adequate time and space for platelet aggregation [30,31]. Moreover, graft remodeling is widely observed in post-operative follow-up visits of direct revascularization, underscoring the importance of this WSS issue when considering graft morphology.

In our study, WSS distribution exhibited significant variability based on the graft angle. Specifically, the 30º angle yielded the largest WSS imbalance across the graft anastomosis, as illustrated in Fig 4. Hence, our result suggests that to achieve a uniform post-operative WSS distribution, the back perfusion of the outlet MV should aim to match the forward perfusion of the inlet MV. As illustrated by our results, how to achieve this would depend on the graft angle and inlet/graft flow rate ratio. Our CFD simulations additionally highlighted areas near the graft branch with markedly increased WSS, which could elicit distal anastomotic intimal hyperplasia and graft failure [32]. WSS in this region is likely modulated by the anastomosis shape and size, suggesting that studies exploring these surgical variables should be explored in future work. Taking WSS into account, despite the 30° angle providing the highest back perfusion, it also produced the most non-uniform WSS distribution. Considering that the majority of patients with MMD are children or in their 30s to 50s [9], long-term outcomes must be carefully considered. Hence, although the 60º case produced slightly less backflow, it would likely yield less remodeling, making it potentially the preferred option. Future work should explore the WSS distributions across remodeled states of the graft morphology.

It is worth noting that CFD consistently reproduced the angle-dependent trends observed in PIV, but with magnitudes ~15–20% lower. This offset likely arises because CFD enforces perfectly steady boundary conditions whereas the peristaltic pumps used in PIV introduce small fluctuations at low flow rates, which can elevate flow reversal. More detailed comparisons between the two modalities is provided in the Supplementary material S1 File. Nonetheless, the preservation of the same angle-ranked patterns across modalities confirms the robustness of our findings.

This study had several limitations. First, the use of PDMS models presents an idealized representation that does not fully capture the intricate structural variations observed in vivo. Specifically, our models did not accurately replicate the MCA with its diverse diameters, non-uniform wall thickness, and tortuosity. Another limitation is that the working fluid used in PIV differed slightly in viscosity and density from the blood properties applied in CFD. To verify the impact of this difference, we conducted a validation CFD simulation using the same fluid properties as in the experiment. The results showed that while absolute WSS increased by ~30%, the velocity fields changed by less than 1%, while the WSS distribution had no changes. Because all flows were laminar and our analysis focuses on comparative velocity and WSS distributions across graft angles, this discrepancy does not alter the relative trends or the main conclusions. Additionally, the variability in donor graft selection—including differences in diameter and shape—could impact hemodynamic outcomes. Another limitation is that our analysis focused on steady flow to examine backflow. Incorporating pulsatile flow could provide further insights into WSS parameters such as oscillatory shear index and relative residence time; future work should explore this. Nevertheless, considering that this is a fundamental study, our results remain valid and provide convincing data. Moreover, our use of stereo-PIV provided planar data, limiting our ability to study three-dimensional velocity and WSS distributions. However, given the relatively simple flow patterns, neglecting the third dimension is unlikely to alter the main conclusions. Therefore, while our results offer valuable insights into the hemodynamic changes associated with different graft angles, additional studies utilizing patient-specific geometry and flow data are needed.

## Conclusion

Our findings suggest that the graft angle in direct cerebral bypass surgery could play a significant role in enhancing backflow. Nevertheless, the increased angle was also associated with a higher disparity in the WSS in the MV, which might promote vessel remodeling and potentially lead to long-term complications. A graft-recipient angle of 60º may best achieve the dual goals of target vascular tree perfusion and long-term bypass construct health. Overall, our study is among the first to consider the impact of bypass construct hemodynamics on surgical decisions. While our results offer a preliminary fundamental reference for surgical decision-making in direct cerebral bypass surgery, further studies are needed to discover the influence of additional surgical variables on a patient-specific basis. Moreover, our findings highlight a need to balance optimal surgical decisions which produce the most preferred immediate outcome against the long-term complications they may promote.

## Supporting information

S1 File PIV and CFD comparison. The CFD model used for this work was validated against corresponding particle image velocimetry (PIV) experimental data. This supplementary file provides the analysis done for this validation step.
(DOCX)
