# Supplementary material for: Evaluating the role of graft angle on cerebral hemodynamics following direct cerebral bypass for moyamoya disease
Source: PLoS One. 2026 Jan 5;21(1):e0330362. doi: 10.1371/journal.pone.0330362 (PMC12768355; doi:10.1371/journal.pone.0330362)
Supplement: S1 File — The CFD model used for this work was validated against corresponding particle image velocimetry (PIV) experimental data. This supplementary file provides the analysis done for this validation step. (DOCX) [file pone.0330362.s001.docx]

**PIV and CFD comparison**

**S1 Fig.** The velocity discrepancy for inlet/graft = 0/10 ml/min (a), 3/10 ml/min(b) and 5/10 ml/min(c). The width of PDF shows relative distribution density. Mean and median values, interquartile ranges, and 95% confidence intervals are indicated.


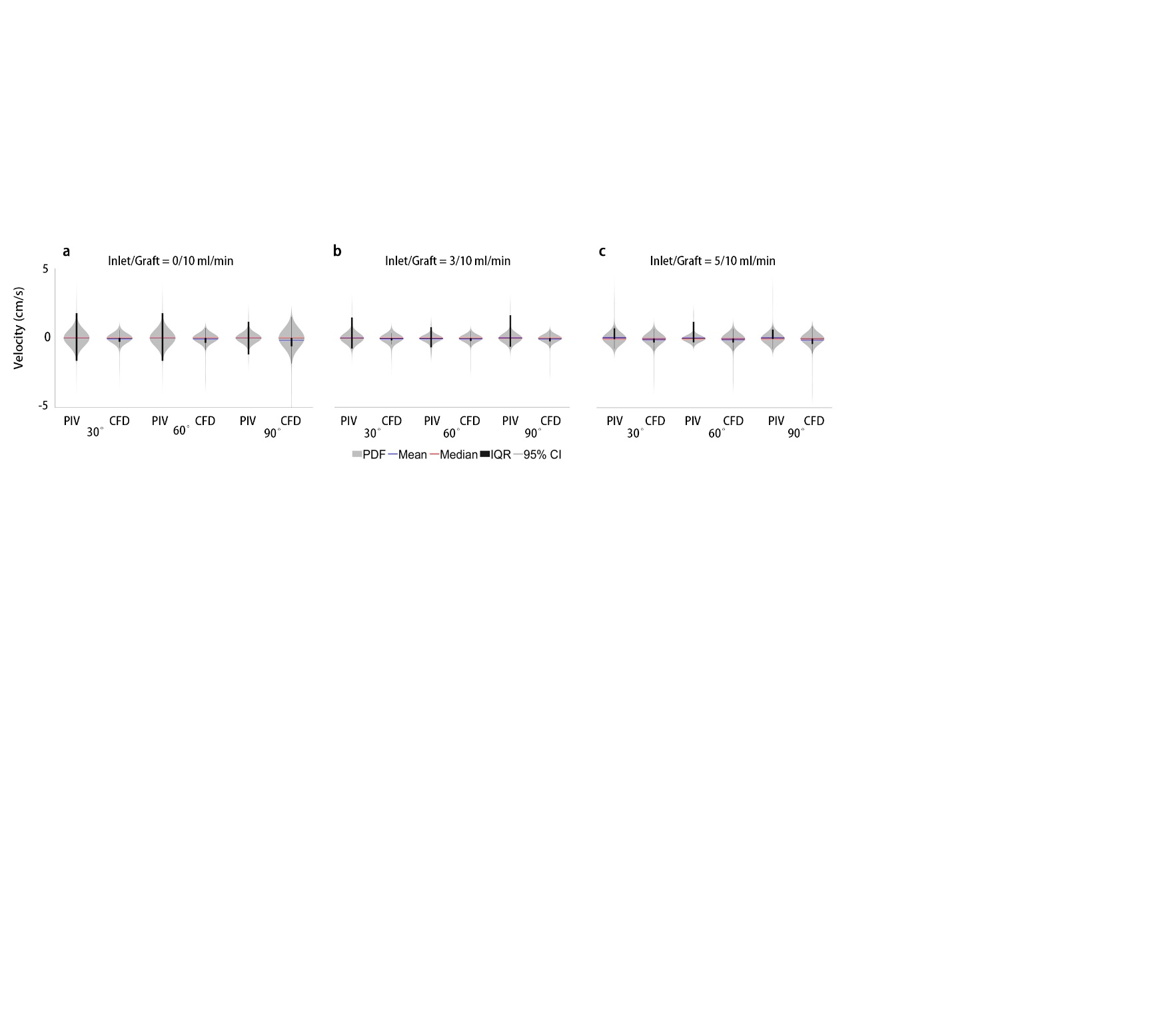


S1 Fig quantitatively compares the velocity discrepancy across the modalities. S1 Fig shows the velocity distribution, interquartile ranges, and 95% confidence intervals for the three angles across three inlet flowrate cases with the same graft flowrate. The “violins” (gray regions) show the overall velocity distribution for each case. In particular, the vertical width of the “violins” represents the range of the data, while the horizontal width represents the data density. The PIV and CFD velocity fields were observed to maintain low discrepancy, particularly for the inlet flow rates of 3 and 5 mL/min. Across all cases, the average velocity discrepancy between CFD and PIV was 2.37 $\times{10}^{-2}$ cm/s, or 7%. All cases had a mean velocity close to 0. The 30° cases had the lowest discrepancy between the PIV and CFD, with only around 0.002 cm/s difference, on average. The 90° had an average discrepancy of 0.0023 cm/s, while the 60° has the highest average discrepancy of 0.007 cm/s. Overall, the discrepancy between the 2 modalities is ~7%.

The two methods showed good overall consistency in both velocity vectors and WSS distributions. This similarity effectively validated the accuracy of CFD simulations. Considering the difficulty of producing some complex *in vitro* models, this validation suggests that simulation studies alone could be used for more comprehensive and complex tests in the future. Fig 2 demonstrates that both modalities yielded similar flow fields, with no significant vorticity or recirculation zones observed near the anastomosis. Notably, previous studies on coronary artery bypass grafts have indicated the presence of small recirculation zones near the junction [1]. This difference can be attributed to the fact that coronary bypass grafts aim to augment normal flow and typically involve much higher flow rates than our case. Fig 4 highlighted the similar WSS distributions across modalities. The PIV WSS distribution was noisier, as expected, since derivative quantities like WSS amplify unavoidable experimental errors. Some significant differences were observed between modalities, at times. Fig 3b and 3c show the only cases in which PIV detects back-flow while CFD does not. The mismatch arises from the mechanical limits of the syringe pumps: synchronizing two pumps at very low rates introduces slight, unsteady fluctuations that PIV records as residual retrograde flow, whereas the CFD model imposes perfectly steady boundary conditions. The flow data collected from flowmeter verified this limitation: for 3 ml/min flow, there is a 109.31% fluctuation, for 5 ml/min flow, such fluctuation reduced to 45.01%, for 10 ml/min flow, it further reduced to 28.12%. Thus, once graft flow rises above this low-range threshold, pump output stabilizes and the two modalities agree. Nevertheless, these low-flow discrepancies do not affect the overall conclusions about how graft angle influences perfusion benefits and shear-related risks.

**References**

1. Liu Z, Yang G, Nan S, Qi Y, Pang Y, Shi Y. The effect of anastomotic angle and diameter ratio on flow field in the distal end-to-side anastomosis. Proc Inst Mech Eng [H]. 2020 Apr 1;234(4):377–86.
